# Regulation of copper uptake by the SWI/SNF chromatin remodeling complex in *Candida albicans* affects susceptibility to antifungal and oxidative stresses under hypoxia

**DOI:** 10.1093/femsyr/foae018

**Published:** 2024-05-17

**Authors:** Inès Khemiri, Faiza Tebbji, Anaïs Burgain, Adnane Sellam

**Affiliations:** Montreal Heart Institute/Institut de Cardiologie de Montréal, Université de Montréal, 5000 Rue Bélanger, Montréal, QC H1T 1C8, Canada; Montreal Heart Institute/Institut de Cardiologie de Montréal, Université de Montréal, 5000 Rue Bélanger, Montréal, QC H1T 1C8, Canada; Department of Microbiology, Infectious Diseases and Immunology, Faculty of Medicine, Université Laval, Quebec City, QC, Canada; Montreal Heart Institute/Institut de Cardiologie de Montréal, Université de Montréal, 5000 Rue Bélanger, Montréal, QC H1T 1C8, Canada; Department of Microbiology, Infectious Diseases and Immunology, Faculty of Medicine, Université de Montréal, Montréal, QC, Canada

**Keywords:** *Candida albicans*, antifungal stress, copper homeostasis, hypoxia, oxidative stress, chromatin remodeling, SWI/SNF complex

## Abstract

*Candida albicans* is a human colonizer and also an opportunistic yeast occupying different niches that are mostly hypoxic. While hypoxia is the prevalent condition within the host, the machinery that integrates oxygen status to tune the fitness of fungal pathogens remains poorly characterized. Here, we uncovered that Snf5, a subunit of the chromatin remodeling complex SWI/SNF, is required to tolerate antifungal stress particularly under hypoxia. RNA-seq profiling of *snf5* mutant exposed to amphotericin B and fluconazole under hypoxic conditions uncovered a signature that is reminiscent of copper (Cu) starvation. We found that under hypoxic and Cu-starved environments, Snf5 is critical for preserving Cu homeostasis and the transcriptional modulation of the Cu regulon. Furthermore, *snf5* exhibits elevated levels of reactive oxygen species and an increased sensitivity to oxidative stress principally under hypoxia. Supplementing growth medium with Cu or increasing gene dosage of the Cu transporter *CTR1* alleviated *snf5* growth defect and attenuated reactive oxygen species levels in response to antifungal challenge. Genetic interaction analysis suggests that Snf5 and the bona fide Cu homeostasis regulator Mac1 function in separate pathways. Together, our data underlined a unique role of SWI/SNF complex as a potent regulator of Cu metabolism and antifungal stress under hypoxia.

## Teaser

Modulation of antifungal stress and copper uptake under hypoxia.

## Introduction

*Candida albicans* is the most prevalent fungal colonizer of humans and it is also the first cause of invasive fungal infections with a mortality rate approaching 40% despite the current therapeutic options (Sellam and Whiteway 2016, Fisher et al. 2020). This yeast thrives on mucosal surfaces, occupying diverse niches characterized by varying and dynamic oxygen levels. As a facultative anaerobe, *C. albicans* grow in both oxygen-rich environments such as skin and oxygen-depleted settings such as internal organs, vagina, gastrointestinal tract, and even tumoral environments (Ernst and Tielker 2009, McKeown 2014, Gan and Ooi 2020). While hypoxia is the prevalent condition within the host, the machinery that integrates oxygen status to tune the growth and the fitness of *C. albicans* remains poorly characterized.

Hypoxia is known to impact different facets of the host–fungus interaction and determines the outcome of the infection. From the pathogen side, hypoxia positively impacts fungal virulence, which can worsen the outcome of the infection. For instance, the invasive filamentous growth of *C. albicans* is enhanced under microaerophilic environments and regulators of this process are essential for host infection (Askew et al. 2009, Desai et al. 2015, Glazier 2022, Henry et al. 2022). As their bacterial analogs, fungal biofilms offer hypoxic environments, which drive the distinctive antifungal-resistant phenotype of this sessile growth state and provide a favorable niche for the flourishing of human pathogenic bacteria (Sellam et al. 2009, Fox et al. 2014, Kowalski et al. 2020). Furthermore, hypoxia promotes the masking of *C. albicans* from the immune cells as a strategy to attenuate phagocytic recognition and uptake (Pradhan et al. 2018). As many fungal metabolic and energetic routes (e.g. sterol biosynthesis and respiration) require oxygen, fungal cells have to reprogram these processes to accommodate the hypoxic lifestyle and sustain their fitness (Butler 2013, Sellam et al. 2014, Burgain et al. 2020). In this regard, recent investigation has underscored the role of the chromatin remodeling complex SWI/SNF as a master regulator that governs the hypoxic metabolic adaptation and fungal fitness *in vivo* (Burgain et al. 2019). Furthermore, transcription factors such as Upc2 and Efg1, which govern the biosynthesis of the oxygen-dependent metabolites, ergosterol and unsaturated fatty acids, respectively, were required for growth under oxygen-depleted environments, emphasizing the importance of tuning metabolism for fungal adaptation to hypoxia (MacPherson et al. 2005, Setiadi et al. 2006, Synnott et al. 2011, Puerner et al. 2023).

So far, only few investigations have assessed the impact of hypoxia on environmental stresses that *C. albicans* and other human fungal pathogens confront at the different colonized niches. These works focused mainly on antifungal stress and the effect of oxygen levels on the sensitivity of *C. albicans* to different classes of standard antimycotics. Overall, hypoxia was found to potentiate the activity of amphotericin B and itraconazole on *C. albicans* (Warn et al. 2004). Our recent study also reported an increased sensitivity of *C. albicans* to other antifungals, including azoles (miconazole and fluconazole) and the echinocandin caspofungin in addition to cell wall and endoplasmic reticulum (ER) stressors under hypoxia (Burgain et al. 2020). The potentiation of azoles and amphotericin B activity by hypoxia could be explained by the fact that ergosterol metabolism, which is the target of these antifungal classes, becomes a rate-limiting process under oxygen shortage, which consequently exacerbates fungal growth inhibition (Burgain et al. 2020). So far, the mechanisms whereby oxygen status is integrated into the stress-responsive machinery in fungi remain elusive.

In the current study, we uncovered that Snf5, a subunit of the chromatin remodeling complex SWI/SNF, provides a nexus for integrating oxygen status and stress response. We found that Snf5 is required to modulate *C. albicans* growth in response to azole and amphotericin B particularly under hypoxia. RNA-seq profiling of *snf5* mutant strain exposed to amphotericin B and fluconazole under hypoxic conditions uncovered a signature that is reminiscent of copper (Cu) starvation. Remarkably, the *snf5* mutant exhibited elevated levels of reactive oxygen species (ROS) specifically under hypoxia, which might explain its increased antifungal sensitivity in this specific condition. Furthermore, our data showed that under hypoxic and Cu-starved environments, Snf5 is critical for preserving Cu homeostasis. Accordingly, supplementing growth medium with Cu or increasing gene dosage of the Cu transporter *CTR1* alleviated *snf5* growth defect and attenuated ROS levels in response to antifungals under hypoxia. Genetic interaction analysis suggests that Snf5 and the bona fide Cu homeostasis regulator Mac1 function in parallel pathways. Our investigation establishes an important role of the SWI/SNF complex as a potent hypoxic regulator of antifungal stress and Cu homeostasis.

## Materials and methods

### Chemicals, fungal strains, and culture conditions

All chemicals used in this study were provided by Sigma–Aldrich (St. Louis, MO, USA). Working stock solutions of Cu(II) sulfate (CuSO_4_; 532 mM), bathocuproinedisulfonic acid disodium salt (BCS; 8 mM), fluconazole (3.3 mM), caspofungin (1 mM), and hydrogen peroxide (H_2_O_2_; 90 mM) were prepared using Milli-Q sterile water. Amphotericin B stock solution were prepared using dimethyl sulfoxide (DMSO; Sigma–Aldrich) at a concentration of 1 mM.

Strains used in this study are listed in Supplementary Table S2. *Candida albicans* strains were routinely grown at 30°C under constant agitation (200 rpm) in YPD medium supplemented with uridine (1% yeast extract, 2% peptone, 2% dextrose, and 50 mg/ml uridine). The Ca*CTR1* open reading frame (ORF) was amplified using the CTR1-OE-F/CTR1-OE-R primer pair and the resulted polymerase chain reaction (PCR) products were cloned in the CIp-Act-Cyc plasmid to generate CIp-Act-*CTR1*-Cyc overexpressing construct (Blackwell et al. 2003). The plasmid was linearized with StuI restriction enzyme and integrated into the SN148 WT (Wild Type) strain or *snf5* mutant strain (DHY3) (Finkel et al. 2012). *snf5 mac1* double homozygous mutant was generated by deleting the two *MAC1* alleles in *snf5* (DHY3) strain using *URA3*- and *SAT1*-deletion cassettes (Schaub et al. 2006). All primers used in this study are listed in Supplementary Table S2.

For growth assays in liquid medium, overnight cultures of *C. albicans* were resuspended in fresh YPD medium at an OD_600_ of 0.1 and added to a flat-bottom 96-well plate in a total volume of 150 µl per well in addition of the tested compounds. For each experiment, a compound-free positive growth control and a cell-free negative control were included. Growth assay curves were performed in a BioTek^™^ Cytation™ 5 plate reader at 30°C under normoxic (21% O_2_) or hypoxic (5% O_2_) conditions with constant agitation. For spot dilution assays, overnight cultures were diluted to an OD_600_ of 0.1 and 5-fold serial dilutions were prepared in distilled water. A total of 4 µl of each dilution was spotted on YPD plates containing drugs. Plates were incubated at 30°C for 2 days under both normoxic (21% O_2_) and hypoxic (5% O_2_) conditions using the Heracell VIOS Tri-Gas incubator and imaged using the SP imager system.

### Expression analysis by RNA-seq and RT-qPCR

Overnight cultures of *snf5* mutant and WT (day 185) strains were diluted to an OD_600_ of 0.1 in 40 ml of fresh YPD medium flushed with nitrogen to maintain a hypoxic environment (5% O_2_) and grown at 30°C under agitation (200 rpm) to an OD_600_ of 0.4. Cultures were then left untreated or exposed to either fluconazole (1 µg/ml) or amphotericin B (0.5 µg/ml), and incubated at 30°C for 30 min. For each condition, a total of two biological replicates were considered for RNA-seq analysis. Cells were then harvested by centrifugation at 3000 × *g* for 5 min and the pellets were quick-frozen and stored at −80°C.

RNA extractions, library preparation, and RNA-seq procedures were performed as previously described (Khemiri et al. 2020). The R package limma was used to identify differences in gene expression levels between treated and nontreated samples (Ritchie et al. 2015). Nominal *P*-values were corrected for multiple testing using the Benjamini–Hochberg method. Differentially expressed transcripts in Supplementary Table S1 were identified using a false discovery rate of 0.1 (fluconazole treatment) and 0.05 (amphotericin B treatment), and 1.5-fold enrichment cutoff. Gene ontology (GO) analysis was performed using the GO Term Finder of the Candida Genome Database (Skrzypek et al. 2017).

For real-time reverse transcription quantitative PCR (RT-qPCR), cell cultures and RNA extractions were performed as exactly as described for the RNA-seq experiment. cDNA was synthesized from 1 µg of total RNA using a High-Capacity cDNA Reverse Transcription Kit (Applied Biosystems). The mixture was incubated at 25°C for 10 min, 37°C for 120 min, and 85°C for 5 min. Two units per microliter of RNAse H (NEB) was added to the reactions and incubated at 37°C for 20 min to remove RNA. qPCR was performed using a LightCycler 480 Instrument (Roche Life Science) with the PowerUp™ SYBR^®^ Green Master Mix (Applied Biosystems). The reactions were incubated at 50°C for 2 min, 95°C for 2 min, and cycled 40 times at 95°C, 15 s; 54°C, 30 s; and 72°C, 1 min. Fold enrichment of each tested transcript was estimated using the comparative ΔΔCt method. To evaluate the gene expression level, the results were normalized using Ct values obtained from actin (*ACT1*, C1_13700W_A). Primer sequences used for qPCR are summarized in Supplementary Table S2.

### Cu quantification

A colorimetric Copper Assay Kit (Cat# MAK127; Sigma) was used to measure intracellular Cu concentrations, using the manufacturer’s protocol. Briefly, *C. albicans* cells were resuspended at an OD_600_ of 0.1 in fresh YPD medium or YPD supplemented with 400 µM BCS and grown overnight at 30°C. Cells pellets were collected and digested overnight at 100°C using 20% nitric acid.

### Measurement of intracellular ROS levels

Intracellular ROS levels were detected using the cell-permeable fluorescent dye 2′,7′-dichlorodihydrofluorescein diacetate (H_2_DCFDA) (Cat# D6883; Sigma). Yeast cells grown to late exponential phase in YPD were pretreated with the indicated compounds for 4 h at 30°C under either normoxia or hypoxia. After a 30-min incubation with 25 µM H_2_DCFDA in the dark, the cells were harvested, washed three times with PBS, and resuspended in the same buffer to 2 × 10^7^ cells/ml. A total of 100 µl of each suspension was transferred to a flat-bottom 96-well microplate and fluorescence was measured using a BioTek Cytation 5 plate reader (*λ*_ex_ = 485 nm; *λ*_em_ = 528 nm).

## Results

### Differential role of hypoxia in sensitizing *snf5* mutant to antifungals

**Figure 1.**
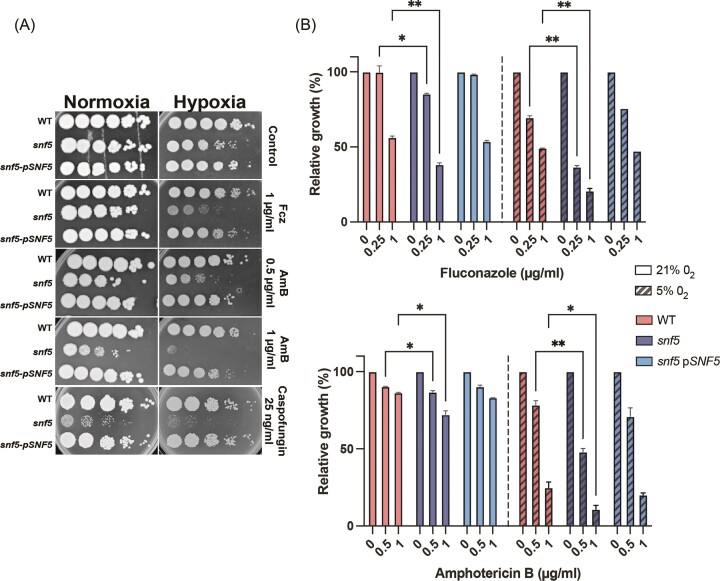
*snf5* exhibits differential antifungal sensitivity under hypoxia. Effect of different classes of antifungals (fluconazole: Fcz, amphotericin B: AmB, and caspofungin) on WT (day 185), *snf5* and *snf5* complemented strain (*snf5*–p*SNF5*) in YPD agar using spot assay (A) or in liquid YPD medium (B) under both normoxia (21% O_2_) and hypoxia (5% O_2_). Results represent mean growth inhibition (%) after 48 h of treatment of at least three replicates. Significance was determined using the two-tailed Student’s *t*-test (*P < .05 and **P < .01).

We have previously uncovered that the SWI/SNF subunit Snf5 is required for metabolic reprogramming to sustain the growth of *C. albicans* under hypoxic environments (Burgain et al. 2019). Here, we tested whether this transcriptional regulator might have an oxygen-dependent role in modulating antifungal sensitivity toward the three-standard class of antifungals represented by fluconazole, amphotericin B, and caspofungin. Serial dilution assays on solid medium showed that *snf5* mutant was hypersensitive to both fluconazole and amphotericin B, specifically under hypoxia as compared to normoxia (Fig. 1A). Nevertheless, *snf5* growth was inhibited by caspofungin irrespective of oxygen levels. Quantitative growth assay on liquid medium confirmed the exacerbated hypoxic defect of *snf5* to both fluconazole and amphotericin B, while only a modest inhibition was perceived under normoxic conditions (Fig. 1B). Together, these observations suggest that the previously characterized oxygen-dependent role of SWI/SNF in metabolic flexibility is expanded to include the modulation of antifungal stress principally under hypoxia.

### RNA-seq profiling of *snf5* in response to both fluconazole and amphotericin B under hypoxic conditions uncovers a transcriptional signature reminiscent of Cu deprivation

**Figure 2.**
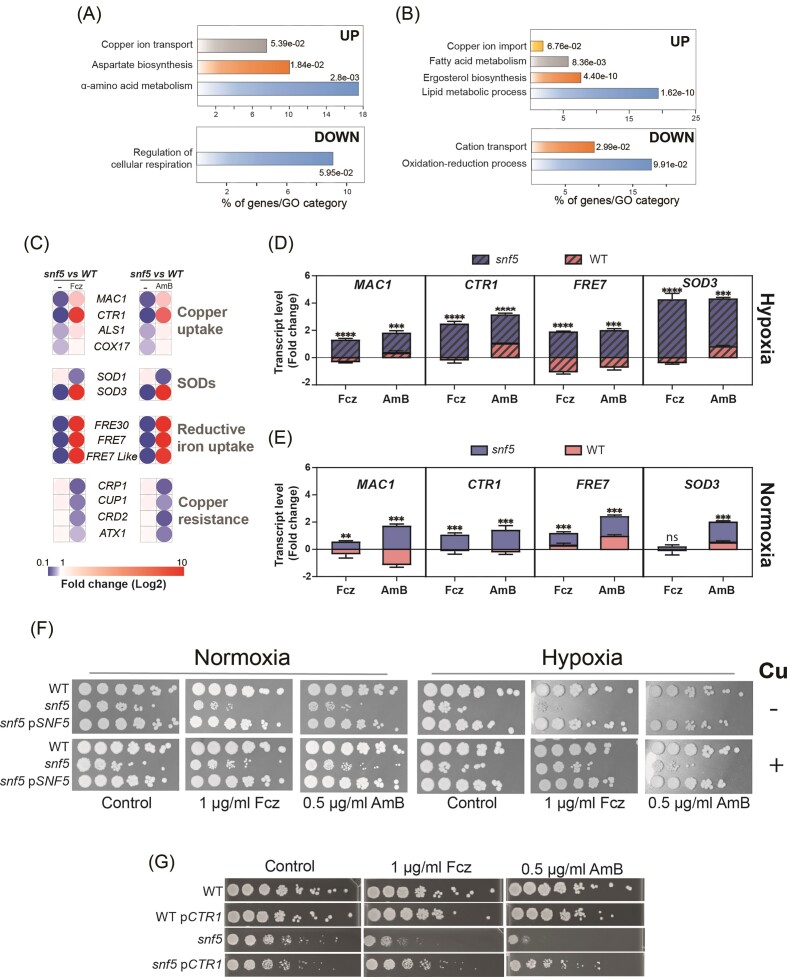
*snf5* sensitivity to antifungals under hypoxia is associated with deregulation of Cu homeostasis. (A and B) Gene functions and biological processes enriched in the transcriptional profiles of *snf5* mutant exposed to either fluconazole (A) or amphotericin B (B). (C) Modulation of Cu regulon by antifungal treatment under hypoxia. Heat map visualization of the transcript levels of the Cu homeostasis pathway in *C. albicans* as defined previously (Khemiri et al. 2020). (D and E) qPCR validation of RNA-seq data. Transcript levels of Cu-utilization genes (*MAC1, CTR1, FRE7*, and *SOD3*) were assessed under both hypoxia (D) and normoxia (E) in response to fluconazole (Fcz) and amphotericin B (AmB). Fold-changes were calculated using the comparative Ct method. Data were normalized using Ct values obtained from the actin gene in each condition. Significance was determined using the two-tailed Student’s *t*-test (**P < .01, ***P < .001, and ^****^P < .0001; ns: nonsignificant). (F) Cu supplementation by 2 mM of CuSO_4_ revert *snf5* susceptibility to antifungals under hypoxia. (G) Increasing *CTR1* dosage in *snf5* alleviates its hypersensitivity to antifungals under hypoxia. Spot assay was used to assess the effect of fluconazole (Fcz) and amphotericin B (AmB) on the growth of WT (ASJC1) and *snf5* (DHY3-CipAct), and their equivalent strains overexpressing the Cu transporter *CTR1* (WT p*CTR1* and *snf5* p*CTR1*).

To understand the contribution of Snf5 in mediating antifungal sensitivity specifically under hypoxia, we performed RNA-seq profiling of both WT and *snf5* cells grown under hypoxic conditions and exposed to fluconazole or amphotericin B. We compared the transcriptional response of *snf5* mutant exposed to each antifungal to that of WT cells treated similarly and found that, for either fluconazole or amphotericin B, Cu-responsive regulon was significantly upregulated in *snf5* (Fig. 2A and B). This includes the Cu transporter *CTR1*, the ferric reductases (*FRE7, FRE30*, and orf19.7077) that reduce Cu to facilitate its uptake by Ctr1, and the master transcriptional activator Mac1 (Fig. 2C). Transcripts associated with Cu efflux and detoxification, including the cytosolic small chaperone *ATX1*, the two metallothioneins *CUP1* and *CRD2*, and the Cu efflux pump *CRP1*, were repressed. We also found that *snf5* mutant upregulated the Mn-dependent superoxide dismutase *SOD3* and repressed the Cu-dependent *SOD1*. qPCR analysis confirmed the RNA-seq data and showed that the modulation of Cu metabolic genes was more marked under hypoxia as compared to normoxia (Fig. 2D and E). This transcriptional signature, reminiscent of a Cu-deprived environment, suggests that *snf5* mutant is unable to maintain a homeostatic level of intracellular Cu upon challenge by antifungals under hypoxia.

Beyond the Cu regulon, genes upregulated in *snf5* exposed to fluconazole were also enriched in aspartate and alpha-amino acid biosynthesis, while genes of lipid metabolism, including fatty acids and ergosterol, were upregulated in response to amphotericin B (Fig. 2A and B). As compared to WT, *snf5* mutant failed to activate genes related to cellular respiration and oxidation–reduction process in response to fluconazole and amphotericin B, respectively.

### Cu repletion revert *snf5* susceptibility to antifungals under hypoxia

To assess whether the perceived antifungal sensitivity of *snf5* under hypoxia is linked to an inappropriate internalization of Cu in *C. albicans*, growth medium was supplemented by 2 mM CuSO_4_. Of note, at 2 mM of CuSO_4_, the growth of *C. albicans* is not altered and it is sufficient to restore growth defect of Cu-uptake mutants as previously shown (Khemiri et al. 2020). Supplementing growth medium with Cu, was sufficient to correct *snf5* sensitivity to fluconazole and amphotericin B under hypoxia at a level comparable to the untreated *snf5* condition (Fig. 2F). Furthermore, overexpressing the Cu transporter Ctr1 reverted the *snf5* sensitivity to antifungal stress under hypoxia to a level similar to that observed in the WT strain (Fig. 2G). Together, these data suggest that the *snf5* exacerbated sensitivity to antifungals under oxygen-depleted environment is a consequence of a perturbed Cu homeostasis.

### Inactivation of *SNF5* results in Cu utilization defect under hypoxia

**Figure 3.**
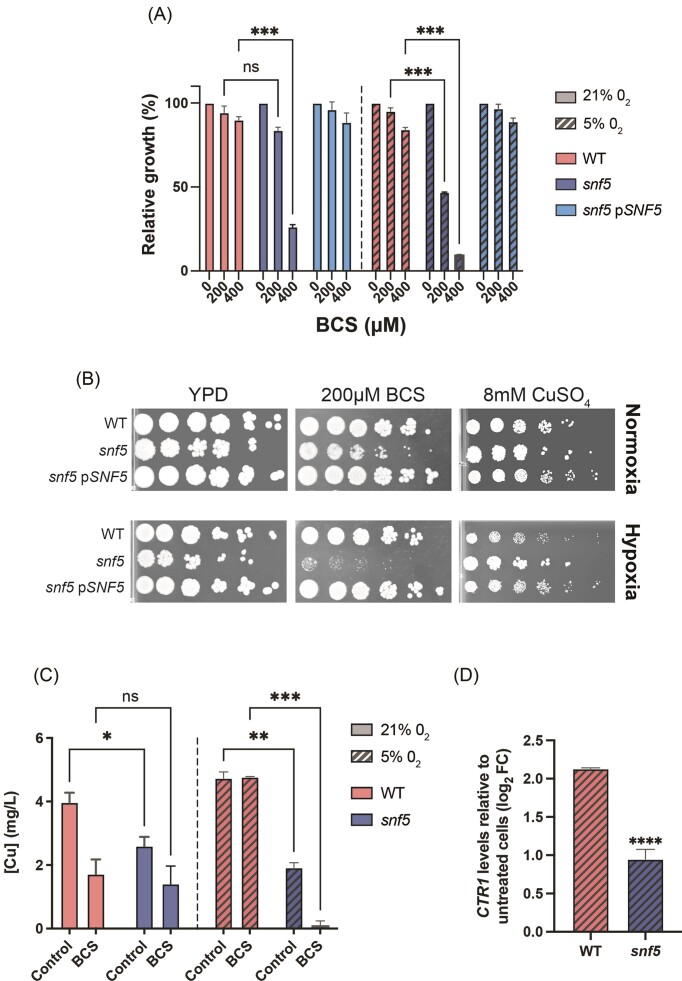
Snf5 is required for Cu homeostasis under hypoxia. Effect of Cu chelation using BCS on the growth of WT (day185), *snf5*, and *snf5* complemented strains (*snf5*–p*SNF5*) in YPD liquid (A) and YPD-agar (B) media. For Cu excess in YPD-agar, cells were treated with 8 mM of CuSO_4_. (C) Impact of *snf5* mutation and Cu chelation (400 µM BCS) on intracellular Cu contents under both normoxia and hypoxia. (D) Transcript levels of *CTR1* assessed by qPCR in WT and *snf5* strains under hypoxia. WT and *snf5* cells were grown in either YPD or YPD supplemented with 400 µM BCS. Transcript levels of *CTR1* were normalized to the untreated condition (YPD). Results represent mean fold changes of at least three replicates. Significance was determined using the two-tailed Student’s *t*-test (*P < .05, **P < .01, ***P < .001, and ^****^P < .0001; ns: nonsignificant).

We and others have previously shown that Cu homeostasis modulates antifungal tolerance in *C. albicans* (Hunsaker and Franz 2019, Khemiri et al. 2020). Accordingly, we hypothesized that Snf5 might govern Cu homeostasis under O_2_-depleted environment, which in turn impacts antifungal sensitivity in this condition. To test this hypothesis, we tested the ability of *snf5* mutant to grow in a medium starved or containing sublethal concentration of Cu. We found that *snf5* mutant exhibited high sensitivity to Cu chelation (BCS) and a better tolerance toward excess of Cu as compared to the WT particularly under hypoxia (Fig. 3A and B). Measurement of Cu content showed that under Cu sufficiency (YPD), *snf5* exhibited more than two order magnitude reduction of Cu as compared to the WT, regardless of oxygen status (Fig. 3C). This suggests that Snf5 is essential for preserving Cu homeostasis in *C. albicans*. When Cu was depleted from the medium using BCS, *snf5* exhibited a similar Cu amount as compared to WT under normoxia. However, under hypoxia, intracellular Cu was almost undetectable in *snf5* as compared to the WT parental strain (Fig. 3C). This underlines that Snf5 has a hypoxic-dependent contribution to Cu homeostasis exclusively under Cu-limiting environments. This is corroborated by the fact that, under Cu scarcity, *snf5* was not able to activate the transcription of the high affinity Cu transporter Ctr1, specifically under hypoxia (Fig. 3D). Overall, these data underscore that Snf5 might integrate both oxygen and Cu availability to tune fungal growth in hypoxic niches.

### Loss of *SNF5* impairs resistance to oxidative stress and increases ROS levels under hypoxia

**Figure 4.**
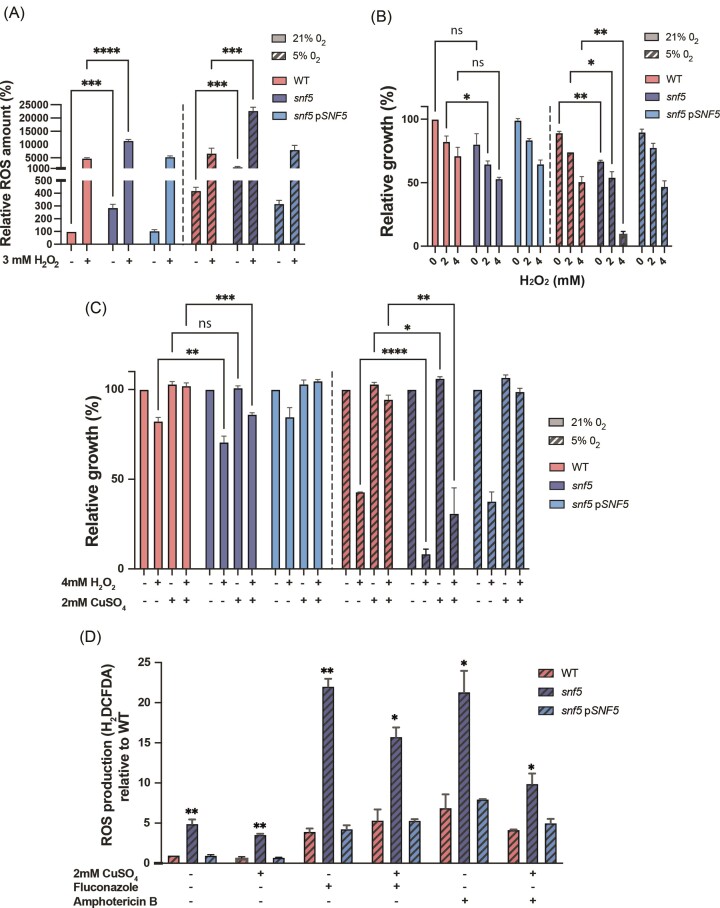
Snf5 modulates oxidative stress under hypoxia. (A) ROS quantification by H_2_DCFDA probe. WT (day 185), *snf5*, and *snf5*-complemented (*snf5–*p*SNF5*) strains were grown under either hypoxia or normoxia in unstressed and stressed (3 mM H_2_O_2_) conditions. Data represent the percentage of ROS levels in the different strains relative to the WT under normoxia. Results are the mean of at least three replicates. (B) Impact of H_2_O_2_ on *snf5* growth under both normoxia and hypoxia. Results represent mean growth inhibition of at least three replicates. (C) Cu supplementation by 2 mM of CuSO_4_ mitigates *snf5* susceptibility to H_2_O_2_ under hypoxia. Strains were grown in liquid YPD medium with or without CuSO_4_ and H_2_O_2_ as indicated. Results are the mean of three replicates. (D) Impact of Cu supplementation (2 mM CuSO_4_) on ROS levels generated by fluconazole (Fcz) and amphotericin B (AmB) treatment in *snf5* mutant. The level of ROS production was measured 4 h after treatment with either 10 µg/ml of fluconazole or 10 µg/ml of amphotericin B under hypoxic conditions. Data represent the mean of three replicates. Statistical significance (WT versus *snf5*) was determined using the two-tailed Student’s *t*-test (*P < .05, **P < .01, ***P < .001, and ^****^P < .0001; ns: nonsignificant).

In addition to their primary targets, antifungals such as amphotericin B and azoles are known to mediate cell death by inducing oxidative damages (Kobayashi et al. 2002, Belenky et al. 2013, Mesa-Arango et al. 2014). We hypothesized that *snf5* might have high basal levels of ROS under hypoxia as compared to normoxia, which might explain the differential sensitivity to the ROS-generating antifungals, azoles and amphotericin B. Notably, Cu is a key cofactor for superoxide dismutases, which are enzymes that promote protection against ROS (Hwang et al. 2002, Frohner et al. 2009). In this regard, as *snf5* has low Cu contents, this might impede Cu-SOD activity, which might lead to the accumulation of high levels of ROS and consequently explain the exacerbated antifungal sensitivity of *snf5* under hypoxia. We found that *snf5* exhibited higher levels of ROS as compared to the WT in either unstressed or H_2_O_2_-treated cells particularly when oxygen was depleted (Fig. 4A). We also tested the impact of *SNF5* inactivation on the sensitivity to H_2_O_2_ and uncovered that *snf5* mutant was hypersensitive to 4 mM H_2_O_2_ specially under hypoxia (Fig. 4B). Supplementation of growth media with Cu mitigated *snf5* sensitivity to oxidative stress, though to a lesser degree compared to the observed alleviation in the WT strain (Fig. 4C). Overall, deletion of *SNF5* leads to higher levels of intrinsic oxidative stress specifically under hypoxia, which might explain *snf5* hypersensitivity to H_2_O_2_. When treated with fluconazole and amphotericin B under hypoxia, *snf5* exhibited higher ROS levels as compared to the WT and the untreated conditions (Fig. 4D). This phenotype was attenuated with Cu supplementation as ROS levels dropped significantly in *snf5* mutant, the WT, and the complemented strains (Fig. 4D). These findings suggest that Snf5 govern antifungal sensitivity under hypoxia by preserving Cu homeostasis to promote Cu-dependent antioxidant response in *C. albicans* cells.

### Functional relationship between Snf5 and the master regulator of Cu homeostasis Mac1

**Figure 5.**
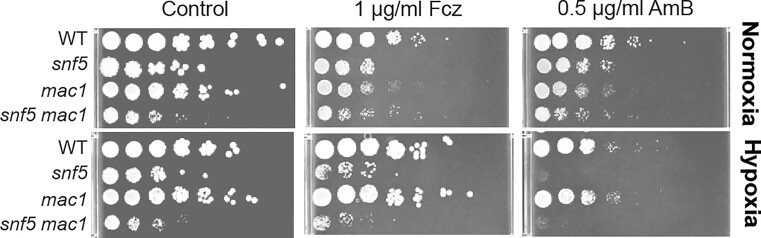
Genetic interaction between Snf5 and Mac1. Additive effect of *snf5* and *mac1* mutations on growth and sensitivity to antifungals. Cultures were grown overnight and spotted on YPD plates with or without antifungals (fluconazole: Fcz; amphotericin B: AmB) as indicated.

We have previously reported that *mac1* mutant was hypersensitive to azoles and amphotericin B under normoxic conditions (Khemiri et al. 2020). As transcription factors require chromatin remodeling complexes to facilitate their accessibility to their target DNA (Burns and Peterson 1997, Neely et al. 2002), we hypothesized that Mac1 and SWI/SNF might operate synergistically to control the transcription of the Cu regulon in *C. albicans*. To test potential epistatic relationship between the two regulators, we deleted *MAC1* in *snf5* mutant and tested the sensitivity of the resulting double mutant to fluconazole and amphotericin B under both normoxia and hypoxia. Overall, under either stressed or unstressed conditions and regardless of oxygen status, *snf5 mac1* double mutant exhibited an additive growth defect as compared to *mac1* or *snf5* single mutants (Fig. 5). This synthetic sickness of *mac1 snf5* indicates that both regulators function in parallel pathways controlling the same biological process. Intriguingly, while *mac1* exhibited hypersensitivity toward the tested antifungals under normoxia as previously reported, this defect was completely buffered by hypoxia.

## Discussion

We have previously found that Snf5 was critical for reprogramming different metabolic routes to accommodate *C. albicans* growth under hypoxic environments (Burgain et al. 2019). In this study, we have uncovered a novel role of Snf5 in mediating stress response and Cu metabolism under hypoxia, which underscores the global role of this regulator in tuning fungal fitness in oxygen-deprived niches.

In metazoans, hypoxia is known to cause ROS accumulation as a consequence of inefficient electron transfer across the mitochondrial electron transport chain (Hamanaka and Chandel 2009, Kung-Chun Chiu et al. 2019). We found that this phenomenon was conserved in *C. albicans* as ROS levels were increased by 3-fold under hypoxia as compared to normoxia (Fig. 4A). Under unstressed conditions, *snf5* accumulated more ROS in hypoxia, which indicates that ROS production exceeds elimination in this mutant. This suggests that Snf5 is required to preserve redox homeostasis and/or to detoxify ROS as an adaptative mechanism to survive in oxygen-depleted niches. Our prior metabolomics analysis of *snf5* cells experiencing hypoxia uncovered reduced levels of metabolic intermediates of the pentose phosphate pathway (PPP), an essential metabolic route for the production of the NADPH that is critical for cellular redox balance (Burgain et al. 2019). Given the importance of PPP for *C. albicans* fitness under hypoxia (Burgain et al. 2020), SWI/SNF might act as a transcriptional regulator of this pathway to promote NADPH supply and accommodate the hypoxic growth. This hypothesis is also supported by our genome-wide occupancy analysis where promoters of the majority of the PPP genes were bound by the Snf2 subunit, suggesting a direct transcriptional control of this metabolic route by SWI/SNF complex (Tebbji et al. 2020). In addition to the potential contribution to the biosynthesis of NADPH, SWI/SNF might modulate oxidative stress indirectly by controlling the uptake of Cu, which is a prerequisite for Cu-dependent SODs to detoxify superoxide radicals. Thus, SWI/SNF might modulate redox status of the cells by means of multiple mechanisms in *C. albicans*.

When challenged by either azole or amphotericin B, *snf5* exhibited a strong growth defect under hypoxia and displayed a transcriptional signature of Cu scarcity. *snf5* mutant has a reduced intracellular amount of Cu and was not able to properly modulate the Cu transporter Ctr1, especially under Cu limitation. Together, these data suggest that SWI/SNF is an important regulator of Cu uptake in *C. albicans*. Cu homeostasis is modulated by the transcription factor Mac1, which is activated by Cu limitation to promote Cu uptake in *C. albicans* (Woodacre et al. 2008, Tebbji et al. 2020). As a chromatin remodeler, SWI/SNF might thus facilitate Mac1 access to their target promoter through nucleosome rearrangement of Mac1-occupied promoters. However, this hypothesis is not supported by the synthetic sick phenotype of *mac1*-*snf5* double mutant, which suggests that Mac1 and Snf5 operate in parallel pathways to preserve Cu homeostasis. Thus, SWI/SNF might cooperate with another transcription factor yet to be discovered or act directly through a different mechanism to modulate the Cu regulon in *C. albicans*.

Intriguingly, *C. albicans* hypoxic cells internalized more Cu than cells growing under normoxic conditions, underscoring the importance of this metal for fungal adaptation in hypoxia. A similar phenomenon was observed in murine macrophages where an increased Cu uptake and redistribution into the secretory pathway in response to hypoxia was perceived (Sarkar et al. 2003, White et al. 2009). This study uncovered that hypoxic macrophages prioritize Cu delivery to post-Golgi vesicles to assist the function of the multicopper ferroxidase in iron (Fe) uptake, underlining that Cu and Fe homeostasis are intimately intertwined. As Cu is also required for reductive Fe uptake in *C. albicans* (Knight et al. 2002, Weissman et al. 2002, Fourie et al. 2018), it is tempting to speculate that a similar mechanism is taking place under hypoxia where Cu demand is increased to sustain the function of multicopper ferroxidase and Fe assimilation.

## Supplementary Material

foae018_Supplemental_Files
